# A Systematic Review on Yellow Fever Vaccine and Adverse Events

**DOI:** 10.1155/ghe3/2016905

**Published:** 2026-07-07

**Authors:** Omar Enzo Santangelo, Anna Sole Pizzamiglio, Vella Carlotta

**Affiliations:** ^1^ Azienda Socio Sanitaria Territoriale di Lodi, ASST di Lodi, Lodi, Italy

**Keywords:** adverse events, review, systematic review, vaccines, yellow fever, yellow fever vaccine

## Abstract

**Introduction:**

Yellow fever (YF) is a hemorrhagic viral infection that can be prevented by a highly effective live attenuated virus vaccine (YFV), which is not free from adverse reactions. This systematic review evaluated the burden of adverse events, including serious ones, due to vaccination.

**Materials and Methods:**

This study was carried out following the guidelines of the Cochrane Collaboration and the Meta‐analysis Of Observational Studies in Epidemiology, PRISMA 2020 checklist, and the Preferred Reporting Items for Systematic Reviews and Meta‐Analyzes.

**Results:**

The suitable bibliography on PubMed/Medline, Scopus, and Web of Science was searched by combining text free, words and titles of medical topics. At the end of the search this systematic review contained 109 records.

**Conclusions:**

The YFV is safe, and serious adverse events are rare although they can be fatal and unpredictable. The most common adverse events are mild even in high‐risk population groups. It is still advisable to assess the appropriateness of administration on a case‐by‐case basis.

## 1. Introduction

Yellow fever (YF) is a viral hemorrhagic infection caused by an RNA virus currently classified within the genus *Orthoflavivirus* (family *Flaviviridae*), according to the latest International Committee on Taxonomy of Viruses (ICTVs) classification. It is closely related to viruses that cause West Nile, St. Louis encephalitis, and Japanese encephalitis and is transmitted to humans through the bite of infected mosquitoes of the *Aedes* or *Haemagogus* species. The virus is endemic in tropical and subtropical areas in South America and Africa [1]. Tree‐hole breeding mosquitoes, such as *Aedes aegypti* and *Haemagogous* species, transmit YF, especially during the rainy season. The “yellow” in the name refers to the jaundice that affects some patients with severe disease. The highest mortality rates are reported in infants and the elderly, who often have depressed immune systems. It can present with varying clinical features ranging from a self‐limited, mild febrile illness, to severe hemorrhage and liver disease. A small proportion of patients who contract the virus can develop severe symptoms and approximately 50% of those die within 7–10 days [2]. Person to person or primate to human transmission has not been reported without the involvement of a mosquito vector, and infected humans may infect mosquitoes during periods of viremia and spread the virus. There is no specific antiviral therapy, but there is an effective vaccine commonly recommended for travelers to at‐risk areas, the infection is prevented by the live attenuated viral yellow fever vaccine (YFV) that are responsible for a significant reduction of disease occurrence [1]. All current YFVs are derived from the 17D lineage: 17DD and 17D‐204 [1]. YF transmission occurs through two main epidemiological cycles: a sylvatic cycle and an urban cycle, involving different mosquito vectors [2, 3]. In the sylvatic cycle, the virus is maintained between nonhuman primates and forest‐dwelling mosquitoes, primarily of the genera *Haemagogus* in South America and *Aedes* species in Africa, with occasional transmission to humans entering forested areas. In the urban cycle, transmission occurs between humans via domestic mosquitoes, mainly *Aedes aegypt* [2, 3]. Recent outbreaks have been predominantly associated with the sylvatic cycle and spillover into human populations rather than sustained urban transmission. However, in settings with low vaccination coverage and high population density, urban transmission mediated by *Aedes aegypti* may occur and facilitate rapid spread [2, 3]. YF vaccine has seroconversion rates of > 95% within 10 days after a single dose. According to the current World Health Organization (WHO) recommendations, a single dose provides lifelong protection, and booster doses are not required; consequently, the International Certificate of Vaccination is valid for the lifetime of the vaccinated individual [3, 4]. The live attenuated YFV is one of the most successful, cost‐effective public health interventions and safest and most efficacious vaccines ever made [4]; however, no vaccine is completely free of adverse effects or the risk of complications; vaccines are often associated with local reactions. Indeed, despite a long history of safe and efficacious YF vaccination, sporadic cases of serious adverse events following immunization (AEFIs) have been reported, with increased serious adverse events in adults who do not have prior immunity given that risk is higher the first time a person is immunized [5]. Among these, we include severe allergic reactions (e.g., egg allergy–associated hypersensitivity reactions, namely, anaphylaxis), neurotropic disease (yellow‐fever vaccine–associated neurotropic disease [YEL‐AND]) and viscerotropic disease (yellow‐fever vaccine–associated viscerotropic disease [YEL‐AVD]). YEL‐AND, formerly known as postvaccinal encephalitis, was the most common AEFIs and YEL‐AVD is especially notable for its lethality, and the pathogenesis of this adverse event is thought to involve uncontrolled replication of the vaccine virus within visceral organs, leading to multiorgan dysfunction [6]. Old reports suggest that AEFIs may have been occurring since the introduction of the vaccine in the 40 and that this trend continues to grow [7]. Therefore, the aim of this systematic review is to evaluate the occurrence, types, and severity of AEFIs after YF vaccination, including serious adverse events where reported.

## 2. Materials and Methods

This systematic review was designed according to internationally recognized methodological standards for observational evidence synthesis, including the Cochrane recommendations and the MOOSE framework [8, 9]. Reporting of the review methodology and findings adhered to the PRISMA 2020 statement [10, 11]. A comprehensive literature search was performed on January 18, 2026, using the Scopus, Web of Science, and PubMed/MEDLINE databases. Search terms were combined through Boolean operators (“AND”, “OR”) to maximize retrieval of relevant studies. The search strategy is reported in Table 1. No time filter was used. To carry out this review, a protocol was created and registered in PROSPERO (international prospective register of systematic reviews) under the number: CRD420261288177.

**TABLE 1:** Full search strategy.

Database	Key words
PubMed/MEDLINE	((“yellow fever vacc^∗^” OR “yellow fever immun^∗^”) AND (“adverse event^∗^” OR “adverse” OR “allerg^∗^” OR “event^∗^” OR “anaph^∗^”))
Scopus	TITLE‐ABS‐KEY (((“yellow fever vacc^∗^” OR “yellow fever immun^∗^”) AND (“adverse event^∗^” OR “adverse” OR “allerg^∗^” OR “event^∗^” OR “anaph^∗^”)))
Web of Science	(((“yellow fever vacc^∗^” OR “yellow fever immun^∗^”) AND (“adverse event^∗^” OR “adverse” OR “allerg^∗^” OR “event^∗^” OR “anaph^∗^”))) Timespan: All years. Indices: Web of Science Core Collection

### 2.1. Inclusion Criteria/Exclusion Criteria

Eligibility criteria were established before study selection. Included studies had to (i) be written in English; (ii) involve human participants, including children, adults, and older individuals; (iii) evaluate YF vaccination; and (iv) report adverse events or serious AEFIs. Observational investigations (cross‐sectional, cohort, and case–control studies), as well as case reports and case series, were considered eligible. Exclusion criteria comprised nonhuman studies, articles not available in English, unavailable full texts, studies unrelated to YF vaccination safety, and publications without data on vaccine‐related adverse events. Reviews, meta‐analyses, editorials, expert opinions, commentaries, conference abstracts, letters, and book chapters were also excluded (see Tables 2 and 3).

**TABLE 2:** Detailed description of inclusion/exclusion criteria, based on Population, Intervention, Comparison, Outcomes, and Study design (PICOS).

Search strategy	Details
Inclusion criteria	P: Human, infant, adult, child, aged (+65) I: Yellow fever vaccine C: Yellow fever vaccine, placebo O: Adverse event, serious adverse event S: Primary observational studies (cohort, case–control, cross‐sectional), case report and case series

Exclusion criteria	P: Not human I: Not about yellow fever vaccine C: Not about yellow fever vaccine and/or adverse event, serious adverse event O: Not about adverse event, serious adverse event S: Review article, meta‐analysis, trial, expert opinion, commentary, editorial, letter to the editor, book chapters.

Language filter	English

Time filter	No filter (from inception)

Database	PubMed/Medline; Scopus; Web of Science

**TABLE 3:** Reasons for exclusion after full‐text assessment.

Author year [ref]	Number of studies	Reasons for exclusion
Centers for Disease Control and Prevention (CDC) 2002 [17]	12	No full text available
Chino et al., 1999 [18]		
Gowda et al., 2004 [19]		
Karouni et al., 2017 [20]		
Kengsakul et al., 2002 [21]		
Merlo et al., 1993 [22]		
Mishra et al., 2018 [23]		
Osinusi et al., 1990 [24]		
Oyelami et al., 1994 [25]		
Pandey et al., 2022 [26]		
Schoub et al., 1990 [27]		
Vital et al., 2002 [28]		

Bruyand et al., 2008 [29]	10	Not in English
Dabrowska et al., 2010 [30]		
Mota et al., 2009 [31]		
Philipps et al., 1996 [32]		
Receveur et al., 2009 [33]		
Solana García et al., 2006 [34]		
Tijssen et al., 2018 [35]		
Traiber et al., 2011 [36]		
Voigt et al., 2001 [37]		
Yaméogo et al., 2009 [38]		

### 2.2. Selection Process and Data Extraction

Screening of titles and abstracts retrieved through the search strategy was independently performed by two reviewers (P.A.S. and V.C.). Potentially relevant studies subsequently underwent full‐text evaluation by the same investigators. Any discrepancies in study selection were resolved through discussion with a third senior reviewer (S.O.E.). Only articles fulfilling all predefined eligibility criteria were retained for qualitative synthesis.

Data collection was carried out using a standardized spreadsheet developed in Microsoft Excel. Extracted variables included author and publication year, study design, country, study period, population characteristics, sample size, reported adverse events, statistical analyses, and additional author comments considered relevant for interpretation.

### 2.3. Strategy for Data Synthesis

**FIGURE 1:**
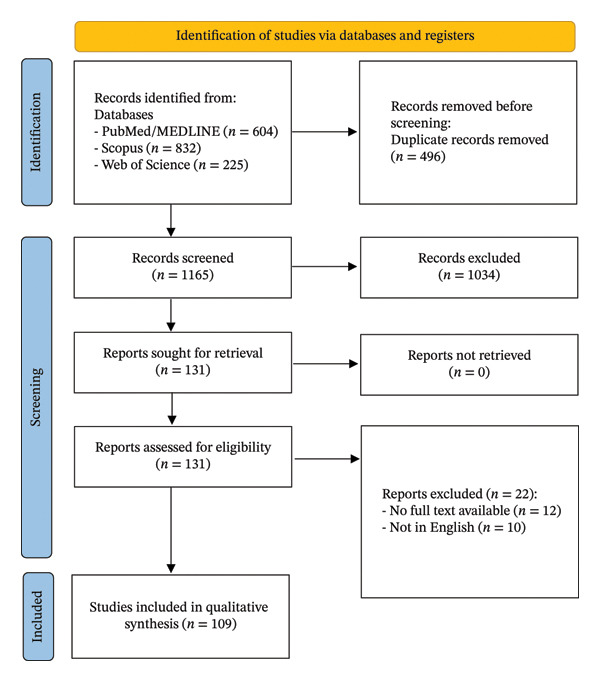
Flowchart of the selection process. No additional records have been identified through other sources.

Study selection was summarized through a PRISMA 2020 flow diagram (see Figure 1) illustrating the number of records identified, screened, excluded, and ultimately included in the review [12]. Descriptive summary tables were also prepared to synthesize the principal qualitative findings of the included studies.

### 2.4. Critical Appraisal

Methodological quality assessment was independently performed by two reviewers (S.O.E. and P.A.S.) using the Newcastle–Ottawa Scale (NOS) for observational studies [13]. An adapted NOS Version was applied to cross‐sectional studies [14]. According to previously adopted thresholds [15, 16], studies scoring ≥ 7 were classified as high quality, scores between 4 and 6 as moderate quality, and scores ≤ 3 as low quality.

## 3. Results

### 3.1. Literature Search

The database search identified 1661 records overall, including 604 from PubMed/MEDLINE, 832 from Scopus, and 225 from Web of Science. After removal of 496 duplicate entries, 1165 records underwent title and abstract screening. Of these, 1034 were excluded because they were not relevant to the topic of interest. Full texts were retrieved for 131 studies, and 22 were subsequently excluded after detailed assessment, mainly because full texts were unavailable or articles were not written in English. Ultimately, 109 studies met the eligibility criteria and were included in the review [22, 39–146]. Reviewer disagreement during the initial screening phase was low (2.9%). Table S1, in supporting information, lists the characteristics of the included studies in alphabetical order by author.

#### 3.1.1. Characteristics of Included Studies

The included literature covered publications from 1983 to 2025 [22, 39–146]. For the sake of brevity, the top three countries in terms of study frequency will be mentioned. For the rest, please refer to Table S2. The studies were conducted in 29 cases in the United States of America [51, 55, 60, 71, 75, 77, 78, 80, 85, 87, 90–93, 96, 99, 100, 102, 105, 107, 109, 115, 118, 124, 128, 130, 141, 144], 26 cases in Brazil [42, 45, 48, 49, 52–54, 56, 63, 68, 82, 94, 95, 97, 98, 101, 103, 108, 112, 114, 119, 122, 132, 134, 137, 139], and 5 in France [62, 76, 84, 117, 143]. Thirty are cross‐sectional studies [43, 47, 56, 63, 66, 69, 72, 73, 75, 77, 80, 82–84, 87, 91–93, 95, 97, 99, 101, 105, 108, 109, 117, 121, 122, 134, 141], 22 are cohort studies [44, 49, 52, 60, 64, 68, 71, 76, 78, 88, 98, 103, 104, 111, 113, 119, 128, 129, 133, 135, 137, 140], and the remainder are case reports or case series. Detailed study characteristics are provided in Table S1.

### 3.2. Quality Assessment of Included Studies

Overall methodological quality was moderate to high across the included observational studies. NOS scores ranged between 6 and 9, with the most frequent limitations involving participant selection procedures and comparability across study groups. A detailed overview of the quality assessment is reported in Table S2.

The following are the results divided by population group.

### 3.3. Children

In the pediatric population, the YFV shows an overall favorable safety profile, with most adverse events being mild and self‐limiting. In a large Brazilian cohort including 3,231,567 children under seven years of age, only 3.9% of the reported adverse events were attributable to the vaccine, mainly including fever, febrile seizures, hypotonic–hyporesponsive episodes, and exanthema [52]. In another analysis of children under 10 years, the most frequent events were generalized rash (24.41%), hypersensitivity reactions (approximately 16%–15%), fever ≥ 39.5°C (12.14%), and fever < 39.5°C (10.21%), while headache and related symptoms accounted for about 3% of the cases [95]. Notably, 70.34% of adverse events occurred in children younger than 1 year [95].

Smaller cohort studies support these findings. In a Korean cohort of 125 children, injection‐site pain was reported in 8.8% of the cases, swelling in 6.4%, redness in 5.6%, fever in 4.0%, headache in 4.0%, cough in 3.2%, abdominal pain in 2.4%, and vomiting in 1.6% [88]. In a study of 435 children with suspected egg allergy, 95.2% experienced no adverse reactions, while 4.8% reported events, mostly mild local or cutaneous reactions, with only one suspected case of anaphylaxis [134]. In a cohort of approximately 2800 individuals during a vaccination campaign, 15 adverse events were identified, of which 13 were nonserious and 2 were classified as serious [113]. Cross‐sectional evidence indicates a low overall incidence of serious adverse events, with a higher frequency of events occurring shortly after vaccination; specifically, 54.2% occurred within 6 h, particularly in younger children [52]. Surveillance data also suggest very low overall rates of adverse events in the general population, estimated at approximately 0.51 events per million doses administered [147–149]. Case series and case reports describe rare but clinically significant events. Three cases of vaccine‐associated neurological disease (YEL‐AND) occurring within 30 days after vaccination have been reported [53], while another series described two cases of viscerotropic disease (YEL‐AVD), both with fatal outcomes [132]. An additional series reported four cases of ocular complications (including both adults and children), generally with favorable outcomes [114]. Pediatric case reports include both mild events, such as fever, irritability, and self‐limiting skin eruptions [79, 94], and more complex neurological manifestations. For example, a 9‐month‐old infant developed neurological symptoms 20 days after vaccination, with complete clinical recovery [112]. Overall, quantitative data indicate that most adverse events in children are mild (generally < 10% for individual symptoms in observational cohorts), while serious events remain extremely rare, although they may include sporadic neurological or viscerotropic cases and very rare fatal outcomes.

### 3.4. Adults

Observational and case report studies show that mild to moderate adverse events—such as myalgia, headache, asthenia, fever, and local reactions—occur in more than 90% of the adult recipients [93, 105, 133, 144]. Severe adverse events, including YEL‐AVD and YEL‐AND, are rare but well documented. The estimated incidence of YEL‐AND is 0.4 per 100,000 vaccines [65], whereas YEL‐AVD cases show variable fatality, with reports of primary vaccine recipients experiencing multiorgan failure or coagulopathy [41, 57, 105, 138, 146]. Mild systemic reactions, including transient fever and malaise, have also been documented, sometimes with bimodal distribution but rarely requiring hospitalization [60]. Rare ocular complications such as MEWDS and uveitis have been described, potentially related to autoimmune mechanisms [42, 131]. Cutaneous reactions, such as pityriasis rosea and cold contact urticaria, have been reported but are uncommon [46, 120, 126, 143]. Overall, the YFV in adults demonstrates high efficacy and safety, with serious events restricted to rare cases.

### 3.5. Elderly (People Aged ≥ 60 Years)

In people aged ≥ 60 years, mild systemic events occur at a rate of approximately 38 per 100,000 vaccinated, with anaphylaxis at 1.8 per 100,000, YEL‐AND at 0.4–0.8 per 100,000, and YEL‐AVD at 0.3–0.4 per 100,000 [39, 40, 55, 58, 59, 85, 89, 96, 103, 105, 106]. Passive surveillance studies associate older age with increased risk of severe events, particularly in those receiving their first dose or with underlying thymic disorders [55, 105, 151]. Reported fatal cases include individuals aged 60–67 with YEL‐AVD and thymic disease [55, 59, 105]. Mild events, such as myalgia, headache, and fever, are common but usually self‐limiting [103]. Herpes zoster infection has also been reported in elderly patients following vaccination [39]. The risk–benefit ratio supports vaccination when exposure risk is high.

### 3.6. People Living With HIV

Small observational studies and retrospective cohorts indicate that YFV is immunogenic and generally safe in HIV‐infected individuals with CD4 counts > 200 cells/mm^3^. No serious AEFIs were reported in these studies [117, 129, 140]. Neutralizing antibody responses were lower compared to noninfected individuals, particularly in the first‐year postvaccination [140]. Higher CD4 counts and suppressed HIV–RNA were associated with better immune response [129]. Serious adverse events appear rare although evidence is limited in advanced HIV infection. Overall, vaccination is recommended for immunologically stable patients traveling to endemic areas.

### 3.7. Pregnancy and Breastfeeding

Observational cohorts of pregnant women, including > 190,000 pregnancies, indicate no increased risk of adverse pregnancy or infant outcomes following YF vaccination [71, 108]. Limited case reports document potential transmission of vaccine virus via breast milk, with rare cases of infant encephalitis [79]. Recommendations emphasize risk–benefit assessment, deferring vaccination if exposure risk is low, or temporarily suspending breastfeeding for ∼10 days postvaccination [79, 147, 148]. Severe adverse events in mothers are uncommon.

### 3.8. Immunomodulatory or Immunosuppressive Therapy

Retrospective and prospective observational studies show that adults on low‐dose or short‐term corticosteroids, DMARDs, or biological therapy tolerate YF vaccination without severe AEFI [31, 72, 76, 149, 150]. Mild to moderate local reactions may occur more frequently under corticosteroid therapy, but systemic severe events are rare. Two fatal events were reported in immunocompromised patients: one in an RA/SLE overlap patient and one in an infant born to a mother under IFX treatment [149]. Sample sizes are generally small, limiting detection of rare severe events.

### 3.9. Thymic Dysfunction

Thymic dysfunction or history of thymectomy is a recognized risk factor for YEL‐AVD. Four fatal cases were reported in individuals with prior thymectomy, aged 44–70 years [149–151]. No further cases have been documented since 2004, possibly due to updated vaccination contraindications.

### 3.10. Multiple Sclerosis

A small case series of seven patients with relapsing‐remitting MS reported increased relapse rates following YF vaccination (annual exacerbation rate 8.57 vs. 0.67 outside the risk period) [149–151]. Vaccination decisions should carefully weigh individual risk of disease exposure versus risk of MS relapse.

### 3.11. Transplant

Studies of solid organ and hematopoietic stem cell transplant recipients suggest that YF vaccination can be tolerated in selected patients without severe immunosuppression [101, 149, 151]. Mild AEFIs, such as nausea, were occasionally reported, but no severe events were documented. Booster doses should be delayed until immune recovery. Clinical judgment is essential for vaccination in this population.

### 3.12. Hypersensitivity to Egg Antigens

Egg‐allergic patients can safely receive YF vaccine using screening and graded administration protocols. Reported incidence of anaphylaxis ranges from 0.42 to 1.8 per 100,000 doses, mostly related to egg protein [68]. Skin testing and intradermal testing allow safe administration even in individuals with recent severe reactions. No severe reactions occurred during carefully monitored protocols [68, 106].

**FIGURE 2:**
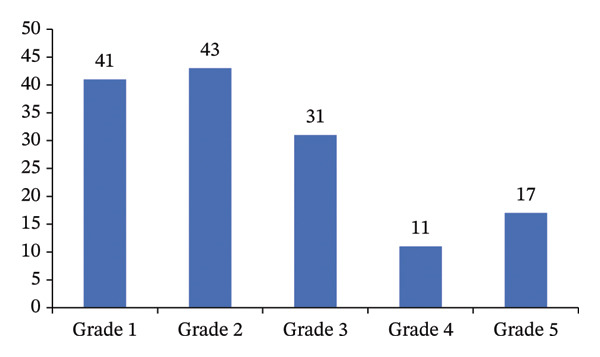
Number of studies in relation to the Common Terminology Criteria for Adverse Events (CTCAEs).

Table 4 shows a summary of the incidence of adverse reactions by population group, only studies that had a denominator among the studies in Table S1 were included in the calculation. Figure 2 shows the number of studies by grade of adverse reaction according to Common Terminology Criteria for Adverse Events (CTCAEs) [152], further details in Table S1. Adverse effects of the YFV can range from mild to severe. Mild to moderate reactions typically include local symptoms at the injection site, such as pain, redness, swelling, induration, and warmth, which usually resolve within one to three days. Mild systemic symptoms may also occur, including moderate fever, headache, muscle and joint aches, fatigue, chills, maculopapular or erythematous rashes, mild nausea, vomiting, diarrhea, and minor neurological events such as transient headache, mild dizziness, or peripheral neuropathy without functional impairment [152]. Severe and potentially fatal adverse events include YEL‐AVD, which presents with clinical features resembling wild‐type YF and can involve multiorgan failure affecting the liver, kidneys, coagulation, and blood pressure, often proving fatal, especially in primary vaccine recipients, older adults, or individuals with thymic dysfunction. Another serious complication is YEL‐AND, characterized by meningoencephalitis, Guillain–Barré syndrome, acute disseminated encephalomyelitis, and bulbar palsy, which can rarely be fatal. Severe allergic reactions, such as anaphylaxis, may occur due to vaccine components and can include urticaria, angioedema, respiratory distress, hypotension, and shock. In extreme cases, severe systemic reactions may require hospitalization and can lead to multiorgan failure, respiratory distress, or fatal complications such as disseminated intravascular coagulation, with death resulting from YEL‐AVD or YEL‐AND in rare instances [152]. As reported in Figure 2, most studies are Grade 1 or 2, indicating that reactions are generally mild/moderate.

**TABLE 4:** Yellow fever vaccine adverse events by population (with incidence).

Population group	Mild/moderate AE (incidence/100,000 doses)	Severe/fatal AE (incidence/100,000 doses)
Children	800–1200	0.83
Adults 18–59 years	3000–10,000	0.3–0.8
Older adults (> 60 years)	5000–12,000	0.3–0.8
People living with HIV	1500–3000	< 1
Pregnancy and Breastfeeding	2000–5000	< 1
Immunomodulatory or immunosuppressive therapy	2000–4500	0.4–2.3
Thymic dysfunction (history of thymoma/timectomy)	Not available	< 1
Multiple sclerosis	2500–3500	< 1 but possible increase in relapses
Transplant	1500–2500	< 1
Hypersensitivity to egg antigens	2000–3000	0.42–1.8 (anaphylaxis)

## 4. Discussion

The overall safety profile of the YFV is widely considered favorable, with the vast majority of AEFIs being mild and self‐limiting, although rare but potentially severe adverse events must be carefully considered in specific populations. In pediatric subjects, available evidence indicates a relatively low incidence of AEFIs, yet age appears to be an important determinant of susceptibility. In particular, 70.34% of the reported adverse events occurred in children younger than 1 year, and among these, generalized rash accounted for 24.41% of the cases [95]. In another cohort, the YFV was responsible for 3.9% of all reported AEFIs in children under 7 years [52], while passive surveillance data estimated a very low overall reporting rate of 0.51 AEFI per million doses [149]. Neurological adverse events remain rare but show a slightly higher incidence in children aged 5–9 years, with a rate of 0.83 per 100,000 doses [97]. Most of these events can be classified according to CTCAEs as Grades 1‐2, reflecting mild to moderate severity, whereas rare neurological or viscerotropic complications, including fatal cases, fall into Grades 3–5 and are likely linked to host‐specific or idiosyncratic responses [139]. In adults, more than 90% of the reported adverse events are nonserious [93], typically presenting within the first week after vaccination with symptoms such as myalgia, headache, asthenia, mild fever, and local reactions [105, 133, 144]. These manifestations correspond predominantly to CTCAE Grades 1‐2. Nevertheless, rare serious adverse events such as YEL‐AND and YEL‐AVD have been documented [77, 146]. The incidence of YEL‐AND is estimated at 0.4 per 100,000 doses, while YEL‐AVD occurs in approximately 0.3–0.4 per 100,000 doses [22]. These conditions are clinically severe, often requiring hospitalization, and, therefore, align with CTCAE Grades 3–5. YEL‐AVD in particular resembles wild‐type YF infection, with cases reporting viremia levels up to 1000 times higher than expected and progression to multiorgan failure and death [41]. Although risk factors such as age over 60 years and thymic disease have been identified, cases in young and apparently healthy individuals suggest that genetic susceptibility and immune dysregulation may also play a role [44, 106]. Surveillance data further indicate that mild systemic symptoms such as fever occur in approximately 3% of the vaccine recipients, often peaking at Day 4 or Day 10 postvaccination, sometimes associated with headache and myalgia [60]. In the elderly population, the frequency of mild adverse events remains relatively low, with 84.7% of the individuals aged 60 years or older reporting no adverse events following vaccination [103]. When present, symptoms such as myalgia, headache, and fever are generally mild and correspond to CTCAE Grades 1‐2. However, the risk of severe adverse events increases with age, particularly for YEL‐AVD, which has an estimated incidence of 0.3–0.4 per 100,000 doses and a mortality rate that may reach up to 66% [105]. Additional reported rates include 38 per 100,000 for mild systemic events, 1.8 per 100,000 for anaphylaxis, and 0.4–0.8 per 100,000 for YEL‐AND. These severe manifestations clearly fall within CTCAE Grades 4‐5. Advanced age, especially in primary vaccine recipients, and underlying thymic abnormalities are key risk factors contributing to these outcomes [55, 151]. In immunocompromised individuals, particularly those living with HIV, available studies—although limited—suggest that the vaccine is generally safe in patients with CD4 counts greater than 200 cells/mm^3^, with no significant increase in serious adverse events reported [117, 129]. However, immunogenicity appears reduced, as HIV‐infected individuals tend to develop lower concentrations of neutralizing antibodies, especially within the first year after vaccination [140]. Higher CD4 counts and suppressed viral load at the time of immunization are associated with better immune responses. Most adverse events in this population are mild (CTCAE Grades 1‐2), but caution remains necessary due to limited evidence in individuals with advanced immunosuppression and the potential for altered immune responses. Special populations such as pregnant and breastfeeding women require a careful and individualized risk–benefit assessment. Although large cohort data involving over 190,000 pregnancies did not show an increased risk of adverse maternal or fetal outcomes following vaccination [71], the use of live attenuated vaccines is generally contraindicated during pregnancy unless the risk of infection is high. In breastfeeding women, rare cases of transmission of the vaccine virus to infants have been documented, including instances of encephalitis confirmed by the presence of YF‐specific IgM antibodies in cerebrospinal fluid [79]. These events, although extremely rare, correspond to CTCAE Grades 3‐4 and highlight the need for caution, particularly in infants younger than 9 months. In patients receiving immunosuppressive or immunomodulatory therapies, current evidence remains limited but suggests that vaccination may be safe under specific conditions, such as low‐dose or short‐term corticosteroid therapy (< 20 mg/day prednisone equivalent), with no severe adverse events reported in small cohorts [31, 72]. However, moderate local reactions appear more frequent, and the limited sample size does not allow exclusion of rare severe outcomes. Similarly, transplant recipients may tolerate the vaccine, but given the theoretical risk of uncontrolled replication of the attenuated virus, vaccination is generally contraindicated in this population despite some reassuring observational data [101]. Hypersensitivity reactions, particularly in individuals with egg allergy, occur at rates ranging from 0.42 to 1.8 per 100,000 doses and are typically attributed to residual egg proteins in the vaccine [68]. Recent evidence suggests that even individuals with confirmed egg allergy can be safely vaccinated using graded administration protocols and appropriate prevaccination testing, with most reactions being mild and consistent with CTCAE Grades 1‐2. This approach minimizes the risk of severe allergic reactions while allowing protection against a potentially fatal infection.

Additional risk factors for severe adverse events include thymic dysfunction and autoimmune diseases. Approximately 17% of the reported YEL‐AVD cases have been associated with a history of thymectomy or thymic disease, reinforcing its role as a contraindication to vaccination [151]. Furthermore, rare autoimmune phenomena such as acute disseminated encephalomyelitis, Guillain–Barré syndrome, neuromyelitis optica spectrum disorder, and ocular inflammatory conditions have been described following vaccination, suggesting possible immune‐mediated mechanisms [42, 127]. These conditions are typically severe and correspond to CTCAE Grade 3 or higher.

From a clinical practice perspective, these findings underscore the importance of a tailored prevaccination assessment aimed at identifying individual risk factors such as age, immune status, thymic history, and comorbidities. In particular, clinicians should carefully evaluate the indication for vaccination in elderly individuals, especially those receiving their first dose, and in patients with known or suspected immunosuppression, balancing the risk of adverse events against the risk of exposure to wild‐type YF virus. Pretravel consultations represent a critical opportunity to stratify risk and to provide informed counseling, including discussion of the rare but severe complications such as YEL‐AND and YEL‐AVD. Moreover, the classification of adverse events according to CTCAE can support clinicians in standardizing the assessment and reporting of vaccine‐related toxicity, facilitating the early recognition of severe reactions and improving comparability across studies and surveillance systems. Active postvaccination monitoring, particularly within the first 10 days when viremia typically occurs, is essential for early detection of warning signs such as persistent fever, neurological symptoms, or signs of organ dysfunction. In high‐risk groups, including breastfeeding women, immunocompromised patients, and those with egg allergy, vaccination should be performed in specialized settings where appropriate monitoring and management of potential adverse reactions are available. Finally, these data highlight the need for ongoing pharmacovigilance and patient education. Clinicians should encourage prompt reporting of AEFIs and contribute to surveillance systems to improve the understanding of rare events. At the same time, clear communication with patients is essential to contextualize the very low incidence of severe adverse events compared to the high morbidity and mortality associated with YF infection, thereby supporting informed decision‐making and maintaining confidence in vaccination programs.

### 4.1. Strength and Limitations

This review presents several important strengths, including adherence to the most up‐to‐date and internationally recognized methodological guidelines for conducting and reporting systematic reviews, which enhances the transparency, reproducibility, and overall quality of the work. Furthermore, it represents one of the most current and comprehensive syntheses of evidence on this topic, incorporating recent data and providing an updated overview of the safety profile of the YFV across different populations.

However, a number of limitations should be acknowledged. First, the findings of this review are inherently influenced by the methodological quality and heterogeneity of the included studies. Many of the original studies rely on passive surveillance systems, which are known to be affected by underreporting, reporting bias, and variability in case definitions, potentially leading to an underestimation of the true incidence of adverse events. In addition, differences in study design, population characteristics, sample size, follow‐up duration, and outcome assessment limit the comparability of results and preclude a fully standardized interpretation of the data. The frequent reliance on observational studies and case reports, especially for rare adverse events such as YEL‐AND and YEL‐AVD, further reduces the ability to establish causal relationships and increases susceptibility to selection and publication bias.

Another limitation is the exclusion of preprint studies, which may have resulted in the omission of relevant and more recent data that have not yet undergone peer review but could contribute to a timelier understanding of the topic. Similarly, restricting the inclusion criteria to articles published in English may have introduced language bias, potentially excluding pertinent studies published in other languages, particularly from endemic regions where YF is more prevalent. Although English remains the dominant language of scientific communication and high‐impact journals, this choice may still have limited the global representativeness of the evidence.

Additional limitations relate to the potential for incomplete data reporting within the included studies. Important variables such as comorbidities, immunological status, prior vaccination history, and detailed clinical outcomes were not consistently available, limiting the possibility of conducting subgroup analyses or identifying specific risk factors. Moreover, variations in adverse event classification systems, with not all studies adopting standardized frameworks such as CTCAE, may have introduced inconsistencies in severity grading and outcome interpretation.

The review is also limited by the absence of quantitative synthesis (e.g., meta‐analysis), which was not feasible due to the heterogeneity of the data and the predominance of descriptive studies. As a result, the conclusions are primarily qualitative and may be subject to interpretative bias. In addition, time‐related biases should be considered, as changes in vaccine formulations, manufacturing processes, and surveillance systems over time may influence the observed safety profile but are not uniformly accounted for across studies. Taken together, these limitations suggest that while the review provides a robust and updated synthesis of available evidence, the findings should be interpreted with caution. Future research should aim to include standardized reporting systems, larger prospective cohorts, and more geographically diverse populations, as well as integrate emerging data sources to improve the accuracy and generalizability of conclusions.

## 5. Conclusions

The YFV remains generally safe, with a very low incidence of serious adverse events. However, although rare, these can be fatal and remain largely unpredictable. Most reported adverse events are mild or moderate in nature, even among population groups considered at higher risk. Despite the high safety profile, it is, therefore, recommended to carefully evaluate the appropriateness of vaccine administration on a case‐by‐case basis, taking into account the individual risk–benefit ratio, comorbidities, and the epidemiological context. This article provides a significant contribution to the scientific community by offering a critical synthesis of the available evidence on the vaccine’s safety and the characteristics of associated adverse events. The data presented can assist researchers in identifying risk factors, improving selection criteria for vaccination candidates, and designing future studies aimed at clarifying the mechanisms underlying rare adverse events. Furthermore, the information discussed may be useful for refining clinical guidelines and postmarketing surveillance strategies, contributing to increasingly safe and informed use of the YFV.

## Author Contributions

Omar Enzo Santangelo: conceptualization, data curation, investigation, methodology, writing–original draft, writing–review and editing, and supervision; Anna Sole Pizzamiglio: data curation, investigation, methodology, writing–original draft, and writing–review and editing; Vella Carlotta: data curation, investigation, methodology, writing–original draft, and writing–review and editing.

## Funding

No external funding was received for conducting this review. The Article Processing Charges (APCs) were covered by the Sistema Bibliotecario Biomedico Lombardo (SBBL), Italy.

## Disclosure

All authors have read and approved the final version of the manuscript.

## Ethics Statement

The authors have nothing to report.

## Conflicts of Interest

The authors declare no conflicts of interest.

## Supporting Information

Additional supporting information can be found online in the Supporting Information section.

## Supporting information


**Supporting Information 1** Table S1. Main characteristics of the included studies. Table S2. Quality assessment of the included studies, using the Newcastle–Ottawa Scale (NOS), reported cohort studies and cross‐sectional studies.

**Supporting Information 2** PRISMA_2020_checklist.

## Data Availability

The data used to support the findings of this study are available on request from the corresponding author.
