# Supplementary material for: A Systematic Review on Yellow Fever Vaccine and Adverse Events
Source: Glob Health Epidemiol Genom. 2026 Jul 7;2026:2016905. doi: 10.1155/ghe3/2016905 (PMC13339928; doi:10.1155/ghe3/2016905)
Supplement: Supplementary file 1 — Supporting Information 1 Table S1. Main characteristics of the included studies. Table S2. Quality assessment of the included studies, using the Newcastle–Ottawa Scale (NOS), reported cohort studies and cross‐sectional studies. [file GHE3-2026-2016905-s002.docx]

**Table S1**. Main characteristics of the included studies.

| **Author/year** | **Study design** | **Population** | **Country** | **Study period** | **Number of partecipants** | **Statistical validation** | **Outcome (adverse events)** | **Comment** | **CTCAE** |
| --- | --- | --- | --- | --- | --- | --- | --- | --- | --- |
| Bayas JM et al. 2007 [39] | Case report | Woman 64 years | Not reported | 2001 | 1 | Not applicable | Brachial herpes zoster infection 3 days after vaccination against yellow fever |  | 2 |
| Beirão P et al. 2017 [40] | Case report | Adults | Portugal | Not reported | 1 | Not Applicable | expression aphasia, agraphia, dyscalculia, right-left disorienta- tion and finger agnosia | The patient had undergone yellow fever vaccination 18 days before the beginning of the symptoms. | 3 |
| Belsher JL et al. 2007 [41] | Case report | 22-year-old femal | Spain | Not reported | 1 | Not Applicable | 22-year-old female died following YF vaccination |  | 5 |
| Biancardi et al. 2019 [42] | Case series | Adults | Brazil | 2018 | 2 | Not Applicable | Anterior and Intermediate Uveitis Following Yellow with Fractional Dose Fever Vaccination |  | 2–3 |
| Biscayart et al. 2014 [43] | Cross-sectional | Persons aged ≥ 9 months | Argentina | 2008-2009 | 1,943,000 | Yes | 165 AEFIs were reported (i.e. 84.9/1,000,000 doses); 35 serious events (i.e.18/1,000,000 doses), including 12 fatal cases, 12 events were classified as YEL-AVD and 12 as YEL-AND. |  | 1–5 (varia, 12 fatal = 5) |
| Breugelmans et al. 2013 [44] | Cohort study | Persons aged ≥ 9 months | Benin, Cameroon, Guinea, Liberia, Mali, Senegal, Sierra Leone, Togo | 2007-2010 | 38,009,411 | Yes | Total AEFIs reporting rate per 100,000 doses= 8.20; Serious AEFI reporting rate per 100,000 doses= 0.43; YF vaccine associated AEFI reporting rate per 100,000 doses= 0.058 |  | 1–2 |
| Brunaldi MO et al. 2021 [45] | Case report | 28-year-old woman | Brazil | 2017 | 1 | Not applicable | Fatal Viscerotropic Disease after vaccination in healty person |  | 5 |
| Brzezinski P et al. 2014 [46] | Case report | Man 30 years | Not reported | 2014 | 1 | Not applicable | 30 years man develope acute onset of pruritic scaly eruptions of the groin, penis, scrotum, and pubic mound after 2 weeks yellow fever vaccination |  | 1 |
| Bühler S et al. 2020 [47] | Cross-sectional | Adults (≥18 years) | Swiss | 2015-2017 | 32 | Yes | 43% of patients and 33% of controls showed local side effects (p=0.71); 86% of patients and 66% of controls reported systemic reactions (p=0.39). First-time yellow fever vaccination was safe and immunogenic in travellers on low-dose methotrexate. | The aim of this study was to compare the safety and immunogenicity of a primary YFVV between travellers on methotrexate and controls. | 1–2 |
| Campos WR et al. 2021 [48] | Case report | A 34-year-old healty male | Brazil | Not reported | 1 | Not Applicable | Vogt-Koyanagi-Harada-like disease after YF vaccination |  | 3 |
| Cavalcanti DP et al. 2007 [49] | Cohort study | Babies>1 month and <1 year | Brazil | Not reported | 304 | Yes | Major malformations (3,3%), minor dysmorphisms, |  | 3–4 |
| Chaves M et al. 2009 [50] | Case report | Adults | Argentina | Not reported | 1 | Not Applicable | progressive paraparesia, urinary retention, and con- stipation. symmetric proximal weakness of the lower extremities. An magnetic resonance (MR) scan of the spine showed longitudinal intra-medullary hyperintense signal | a high concentration of YF vaccine anti- body was found in the CSF. The diagnosis after the observations was for longitudinal myelitis without encephalitis associated with YF vaccine. | 3 |
| Cohen M et al. 2022 [51] | Case report | 67-year-old woman | USA | Not reported | 1 | Not Applicable | Cases of YEL-AND have presented as acute inflammatory demyelinating polyneuropathy, acute disseminated encephalomyelitis, and meningoencephalitis. |  | 3 |
| Cunha MP et al. 2013 [52] | Cohort study | children of less than seven years of age | Brazil | 1998 - 2008 | 3,231,567 | Yes | 20 (3.9%) of the reported AEFI; hypotonic-hyporesponsive episodes (HHEs), fever >39.5°C, febrile convulsion, and generalized exanthema; local reactions with reddening, pain, and induration; onvulsions without fever, and 69.5% of fever (≤39°C) |  | 1–2 |
| de Andrade Gandolfi F et al. 2023 [53] | Case series | 4-year-old female; 10-months-old female; 9-months-old female; | Brazil | 2021 | 3 | Not Applicable | three cases of YEL-AND within the 30-day period following YF vaccination |  | 3 |
| de Filippis et al. 2004 [54] | Case series | Adults | Brazil | 2001 | 2 | Not Applicable | high fever, epigastric tenderness, generalized myalgia, vomiting, icterus and prostration, exanthem, drowsiness, mental confusion, dyspnea and exciting, anuria, oliguria and tachycardia, death. | the two Cases originally identified in the context of a mass vaccination campaign and reported as potential cases of serious adverse events due to the YF 17DD virus vaccine were in fact due to wild type virus infections. | 4–5 |
| DeSilva M et al. 2015 [55] | Case report | Woman 60 years | USA | 2104 | 1 | Not Applicable | 60-year-old female died following YF vaccination |  | 5 |
| Distenhreft JIQ et al. 2024 [56] | Cross-sectional | Individuals with chronic kidney disease | Brazil | 2020-2021 | 223 | Yes | No serious VAE occurred in any patient. Local reactions were reported in 13 individuals (5.8%), while 6 (2.7%) reported generalized systemic reactions and 205 (91.9%) |  | 1 |
| Doblas et al. 2006 [57] | Case report | Adults | Spain | 2004 | 1 | Not Applicable | Yellow fever vaccine-associated viscerotropic disease | Death of patient | 5 |
| Domingo C et al. 2020 [58] | Case report | 74-year-old male | Germany | 2017 | 1 | Not Applicable | nausea, vomiting, diarrhea, general malaise, multiorgan failure, septic shock, |  | 4 |
| Douce RW et al. 2010 [59] | Case report | 67-year-old man | Ecuador | 2008 | 1 | Not Applicable | Febrile illness, headache, severe abdominal pain, anxiety, nausea and vomiting, dyspnea, jaundice, leucopenia, thrombocytopenia, YFV-associated VD |  | 4 |
| Durbin et al. 2009 [60] | Cohort study | Healthy adults | USA | 2005-2007 | 1.341 | Yes | The incidence of fever 3%, headache 2.8%, and myalgia 2.4%. | Monitoring vaccinees with automated telephone/Internet-based system | 1 |
| Ekenberg C et al. 2016 [61] | Case report | 56-year-old woman with Crohn’s disease | Zanzibar, Tanzania | 2015 | 1 | Not Applicable | Following vaccination, the patient fell ill with influenza-like illness. Elevated transaminase levels and YF viremia were detected. Despite being immunocompromised, the patient did not develop more severe adverse effects. | Limited data is available on outcomes of YFV in patients receiving immunosuppressive therapy, including biologics, and we report this case as a reminder of vigilance of vaccine recommendations in this population. | 2 |
| El Nawar R et al. 2018 [62] | Case report | 61-year-old male | France | Not Reported | 1 | Not Applicable | neurotropic disease | Neurological adverse events after yellow fever vaccination are rare or underestimated. To our knowledge, this is the first reported case of meningomyeloradiculitis after yellow fever vaccination. | 3 |
| Facincani et al. 2016 [63] | Cross-sectional | Hemodialysis patients | Brazil | 2012-2014 | 130 | Yes | 24.4% experiencing mild local adverse effects and 4.4% experiencing fever. No serious adverse effects attributable to YF vaccine |  | 1 |
| Fitzner J et al. 2004 [64] | Cohort study | Residents of Abidjan > 9 months of age | Ivory Cost | 2001 | 2,600,000 | Not reported | injection site abscess, severe local reaction, acute encephalitis, convulsions, severe allergic reaction, anaphylaxis, toxic shock syndrome, fever |  | 3–4 |
| Florczak-Wyspiańska J et al. 2017 [65] | Case report | 39-year-old Caucasian man | Poland | Not Reported | 1 | Not Applicable | neurotropic disease | a case of YEL-AND with meningitis presentation in a man without evidence of significant risk factors, which was confirmed by the presence of the YF virus and specific immunoglobulin G (IgG) antibodies in the cerebrospinal fluid (CSF) | 3 |
| García-Paba MB et al. 2023 [66] | Cross-sectional | Persons with history of egg allergy | Colombia | 2017-2019 | 71 | Yes | 2 out of 71 people showed an allergic reaction to the vaccine after vaccination. |  | 2 |
| Gerasimon G et al. 2005 [67] | Case report | 22-year-old female | USA | 2004 | 1 | Not Applicable | Fever, headache, nausea, and vomiting, multiorgan failure, yellow fever vaccineassociated viscerotropic disease (YEL-AVD) |  | 4 |
| Gerhardt CMB et al. 2020 [68] | Cohort study | patients with confirmed egg allergy | Brazil | 2018-2019 | 58 | Yes | hypersensivity reaction in patient showed positive to intradermal test for YFV |  | 2 |
| Gnanadurai R et al. 2022 [69] | Cross-sectional | Travellers | UK | 2016-2018 | 17.520 | Yes | None SAE | The manuscript briefly discussed adverse events, which were comparable in percentage to those listed in the drug's technical data sheet; the article focused more on administration errors | 1 |
| Goldstein EJ et al. 2019 [70] | Case report | Adults | Scotland | Not reported | 1 | Not Applicable | sixth cranial nerve palsy, confusion, fever and expressive dysphasia | had received yellow fever (YF) vaccination 18 days prior to onset of headaches. Group 2 consisted of 10 HIV–individuals from Minnesota, USA, who had been recruited into another protocol. Group 3 consisted of 10 HIV– participants in a study (Yellow Fever Vaccine and Immune Globulin Study; NCT00254826) at Emory University in Atlanta, GA (39), who received YFV with PB collected before vaccination and again 10–14 days later (but no LNs were obtained), providing a group in the U.S. for comparison of immunologic and inflammatory responses elicited by YFV. | 2–3 |
| Hall Cet al. 2020 [71] | Cohort study | population of pregnant military women | USA | 2003–2014 | 196,802 pregnancies and 160,706 singleton infants | Yes | No increased risks for adverse pregnancy or infant outcomes |  | 1 |
| Huber et al. 2018 [72] | Cross-sectional | Patients on immunosuppressive/immunomodulatory therapy | Switzerland | 2008-2015 | 92 | Yes | - | It is not possible to identify the rate of adverse reactions for yellow fever vaccination in this article, rates are general for all live attenuated virus vaccines. | 1 |
| Ishaku SG et al. 2021 [73] | Cross-sectional | General population | Nigeria | 2018-2019 | 504.414 | Not applicable | AEFI vaccines reaction rates/10000 doses: 0.734 | Incidence of AEFI shown but type not reported | 1 |
| Jääskeläinen AJ et al. 2014 [74] | Case report | 50-year-old previously healthy male | Finland | 2014 | 1 | Not Applicable | The YF-vaccine associated neurological disease manifestations include fever, headache, focal neu-rological findings, mental status changes and seizures caused bycentral nervous system tropisms of the vaccine virus itself | We report neurological symptoms after YF-vaccination in a previously healthy Finnish male. Other concomitant infections or causes for the symptoms could not be identified. | 3 |
| Kelso JM et al. 1999 [75] | Cross-sectional | Adults | USA | 1990-1997 | 243 | Not Applicable | 243 VAERS forms reviewed, 40 (16%) describe probable or possible anaphylactic events after the administration of YF vaccine |  | 2–3 |
| Kernéis et al. 2013 [76] | Cohort study | Adults receiving systemic corticosteroid therapy | France | 2008-2011 | 131 | Yes | No serious AEs were reported. The most frequent nAEs reported by the participants were fatigue (n=23), joint pain/myalgia (n=23), headaches (n=19), and pain at the site of vaccine injection (n=13). However, moderate/severe local AEs were experienced 8 times more frequently in participants receiving corticosteroids. No difference was observed for systemic AEs |  | 1–2 |
| Khromava et al. 2005 [77] | Cross-sectional | Persons aged ≥ 9 months | USA | 1990-2002 | 3,157,745 | Yes | Civilian sector: Serious AEFIs reports 1.6/100,000 doses; YEL-AVD reports 0.3/100,000 doses; YEL-AND reports 0.4/100,000 doses. U.S. Military: Serious AEFIs reports 8.6/100,000 doses. | Vaccine Adverse Event Reporting System (VAERS) reports of events followingYELvaccination during 1990–2002, in U.S. or in U.S. citizens stationed overseas | 3 |
| Kityo C et al. 2018 [78] | Cohort study | HIV+ | USA, Uganda | Not reported | 50 | Yes | fibrosis in LNs | Group 1 included 30 HIV– Ugandans who were assessed for baseline measures of inflammatory cytokines in blood and measures of IA in blood and LNs. A subset of 20 of this group was then given YFV with follow-up sampling of peripheral blood (PB) and LNs 10–14 days after vaccination | 2 |
| Kuhn S et al. 2011 [79] | Case report | A five-week-old male infant | Canada | Not reported | 1 | Not Applicable | Fever, irritability, poor appetite, vomiting, rhinorrhea and cough |  | 2 |
| Kuntz et al. 2018 [80] | Cross-sectional | Persons aged ≥18 years | USA | 1997-2007 | 76.606 | Yes | 1 pericarditis | This article analyzes only cases of myocarditis and pericarditis, 1 case of pericarditis during co-administration of yellow fever vaccine with polio, mmr and zoster vaccines. | 3 |
| Lamson DM et al. 2014 [81] | Case report | Woman 19 years | Not reported | Not reported | 1 | Not Applicable | 19 years woman develope ILI after yellow fever vaccination |  | 2 |
| Lara AN et al. 2021 [82] | Cross-sectional | Immunocompromised persons | Brazil | 2017-2018 | 381 | Yes | The most common AE was pain at the injection site (41 persons, 12%), myalgia (34; 10%), fever (25; 7.3%) and headache (16; 4.7%). No Severe AE related to the vaccination. |  | 1–2 |
| Lawrence et al. 2004 [83] | Cross-sectional | Persons aged ≥15 years | Australia | 1993-2002 | 210.656 | Yes | 42 reports of adverse events following Yellow Fever vaccination in the ADRAC (Adverse Drug Reactions Advisory Committee)database that met the criteria for review were identified. Of these, 26 (62%) met the study definition of a 'systemic' adverse event with nine (21%) defined as 'serious systemic' adverse events. One of these nine had died of YFV-AVD | This article only analyzes severe cases, hospitalized cases and deaths | 3–5 |
| Le Hir A et al. 2024 [84] | Cross-sectional | General population | France | 2012-2022 | Not reported | Yes | During the observation period, 10 cases of YEL-AND and 2 cases of YEL-AVD were reported, with an overall incidence of 0.6 per 100,000 doses. A total of 6/12 cases were previously healthy patients (50%, mean age 31 years) and 4/12 cases had muscle comorbidities (42%, mean age 56 years). | Study conducted using data from the French National Reference Centre for Arboviruses (NRCA) over the last 10 years | 3–5 |
| Leal JE et al. 2010 [85] | Case report | 77-year-old Caucasian male | USA |  | 1 | Not Applicable | fever, acute renal failure, hepatitis, lymphocytopenia, thrombocytopenia, hypotension, and respiratory failure. |  | 3 |
| Lecomte E et al. 2020 [86] | Case report | 56-year-old Caucasian man | Belgium | 2018 | 1 | Not Applicable | Fever, headache, cognitive problems, anorexia, nausea with vomiting, flu-like illness |  | 2 |
| Ledlie S et al. 2022 [87] | Cross-sectional | General population | USA | 2004-2019 | 263.979 | Yes | Incidence proportion of neurotropic disease ranged from 0 to 3.04 per 100,000 vaccinees | The article showed data from 3 different databases where serious adverse events are reported. | 1–2 |
| Lee JY et al. 2009 [88] | Cohort study | hildren | South Korea | 2007-2008 | 125 | Yes | pain (8.8%), swelling (6.4%) and redness (5.6%) at the injection site; mild fever (4.0%), headache (4.0%), cough (3.2%), abdominal pain (2.4 %), and vomiting (1.6%); |  | 1 |
| Leung WS et al. 2016 [89] | Case report | Adults | Hong Kong | 2014 | 1 | Not Applicable | fever, loss of appetite, headache and mild myalgia, lethargy and jaundice, deranged liver function tests, elevated creatinine kinase |  | 2 |
| Levy S et al. 2002 [90] | Case series | Adults | USA | 2001-2002 | 6 | Not Applicable | Adverse events after Yellow fever vaccination |  | 2 |
| Liang G et al. 2025 [91] | Cross-sectional | People who have had an adverse reaction to YF vaccine | USA | 2015-2025 | 1664 | Yes | 82.9% of adverse events were non-serious, and 17.1% were classified as serious. Twenty-five of these events resulted in patient death, and seven resulted in permanent disability. In most cases, the primary event was fever and pain at the injection site (approximately 10% of cases). |  | 1–5 (25 deaths = 5) |
| Lindsey et al. 2008 [92] | Cross-sectional | Persons aged ≥ 9 months | USA | 2000-2006 | 1,534,170 | Yes | All AEs 43/100,000 doses; Non-serious adverse events 38.3/100,000 doses; Serious AEs 4.7/100,000 doses; Anaphylaxis 1.8/100,000 doses; YEL-AVD 0.4/100,000 doses; YEL-AND 0.8/100,000 doses. | Reports of adverse events following YF vaccination reported to the U.S. Vaccine Adverse Event Reporting System (VAERS) | 1–4 |
| Lindsey et al. 2016 [93] | Cross-sectional | Persons aged ≥ 9 months | USA | 2007-2013 | 2,224,790 | Yes | All AEs 42.4/100,000 doses; Non-serious adverse events 38/100,000 doses; Serious AEs 3.8/100,000 doses; Anaphylaxis 1.3/100,000 doses; YEL-AVD 0.3/100,000 doses; YEL-AND 0.8/100,000 doses. | Reports of adverse events following YF vaccination reported to the U.S. Vaccine Adverse Event Reporting System (VAERS) | 1–4 |
| Lise MLZ et al. 2018 [94] | Case report | Children | Brazil | Not reported | 1 | Not Applicable | After four days, erythematous papules appeared on palms, soles, trunk and limbs, including macular lesions on the conjunctiva | The lesions dis- appeared in three days without any treatment | 1 |
| Lopes et al. 2018 [95] | Cross-sectional | Persons aged <10 years | Brazil | 2005-2010 | Not reported | Yes | 8.5% of AEFIs per 100,000 doses. Reported 886 AEFIs if single inoculation, 1009 if combined inoculation. About the reported AEFIs 24.41% showed generalized rash, 16.41% hypersensibility reaction up to 2 hours, 15.59% hypersensibility reaction after to 2 hours, 12.14% Fever ≥39.5 °C, 10.21% Fever < 39.5 °C, 4.83% Pain, redness, and heat, 4.14% Meningitis, 3.31% Headache, 3.17% Headache and vomiting |  | 1–2 |
| Martin et al. 2001 [96] | Case series | Persons aged ≥ 63 years | USA | 1996-1998 | 4 | Not Applicable | 3 deaths, clinical features: fever, confusion, hypotension, renal and respiratory failure, rhabdomyolysis. |  | 5 |
| Martins Rde et al. 2014 [97] | Cross-sectional | Adults | Brazil | 2007-2012 | 31,434,631 | Yes | Among the 67 adverse events, 55 were neurotropic (82.1%), and 10 wereneurological autoimmune diseases (14.9%), and 2 were combined disease (3%). Rate of neurological adverse events is 0.20 per 100,000 doses in national analysis. | Report and analysis of 67 neurological cases in Brazil. This study is based on YFV-17DD neurological adverse eventsreported in public health units in Brazil from 2007 to 2012 | 1–3 |
| Martins RDM. et al. 2010 [98] | Cohort study | Population | Brazil | 2000-2008 | 108,000,000 | Yes | Hypersensitivity events (0.9/100 000 doses), YFV-associated ND (0.084/100 000 doses), VD cases (0.019/100 000 doses) |  | 1–3 |
| McClenathan BM 2024 [99] | Cross-sectional | General population | USA | 1999-2018 | 132 | Yes | The incidence rate of YF-VAX vaccine-associated anaphylaxis is estimated at 14.6 events per 1 000 000 doses. |  | 2 |
| McMahon AW et al. 2007 [100] | Case series | Adults | USA | 1990-2005 | 97 | Not Applicable | Six cases of Guillain-Barre Syndrome (GBS), one of encephalitis, and two of ADEM, were classified as ‘suspect’ vaccine-associated disease. |  | 3 |
| Merlo C et al. 1993 [101] | Case report | Adults | Switzerland | 1991 | 1 | Not Applicable | Encephalitis |  | 3 |
| Miranda LJC et al. 2020 [102] | Cross-sectional | renal transplant recipients | Brazil | Not reported | 116 | Not Applicable | 1 vaccinated patient reportedan adverse event (nausea) |  | 1 |
| Miravalle A et al. 2009 [103] | Case report | Man 23 years | USA | Not reported | 1 | Not Applicable | 23 years man with acute disseminated encephalomyelitis after yellow fever vaccination |  | 3 |
| Miyaji et al. 2013 [104] | Cohort study | Persons aged ≥60 years | Brazil | 2009-2010 | 700 | Yes | 15.3% reported AEs, 13.9% had systemic AEs (mainly myalgia, headache and fever) and 2.4% reported AEs at the injection site (1% had both systemic and injection site AE) |  | 1–2 |
| Miyazato Y et al. 2022 [105] | Cohort study | General population | Japan | 2018-2019 | 11.279 | Yes | AEs were observed in 696 participants (6.17%), of which 86 and 656 developed local and systemic AEs, respectively. Immediate AEs occurred in 32 participants. Spontaneous PRO was collected through ePRO from 543 (4.81%) participants. Among these AEs, fever was most frequently observed (424, 3.76%), followed by fatigue (367, 3.25%) and headache (254, 2.25%). Other reportable events, including YEL-AND and YEL-AVD, did not occur. No anaphylactic reactions requiring hospitalization were observed. Two SAEs: tonsillitis and reactive polyarthritis |  | 1–2 |
| Monath et al. 2005 [106] | Cross-sectional | Healthy adults | USA-UK | 2000-2001 | 4.532 | Yes | Clinical trial Protocol H-070-005: Combined ARILVAX and YF-VAX 18-44 years: Body as a whole 67.7%, Digestive System 33%, Muscoskeletal System 24.0%, Respiratory System 3.9%, Skin and Appendages 2.7%. Combined ARILVAX and YF-VAX >64 years: Body as a whole 51.9%, Digestive System 0%, Muscoskeletal System 14.8%, Respiratory System 4.9%, Skin and Appendages 4.9%. Clinical trial Protocol H-070-008: Combined ARILVAX and YF-VAX 18-44 years: Ashtenia 49.8% , Malaise 39.7%, Fever 18.6%, Headache 47.6%, Myalgia 35.4%, Injection site pain 35.9%, Diarrehea 29.6%. Combined ARILVAX and YF-VAX >64 years: Ashtenia 35.2% , Malaise 27.8%, Fever 11.9%, Headache 38.1%, Myalgia 28.4%, Injection site pain 9.7%, Diarrehea 25.0% | Retrospective analysis of two RCT | 1–2 |
| Muñoz J et al. 2008 [107] | Case report | Adults | Spain | 2005 | 1 | Not Applicable | fever (38.7°C), arthro-myalgia, supraventricular extrasystolia, hematuria, and mild jaundice; a petechial rash affecting his legs, axillae, and palate mucosa; flebite e orchite; a mild elevation of liver enzymes were detected; a minimum bilateral pleural effusion. | unusually high levels of specific YFV antibodies shortly after vaccination | 2 |
| Nash ER et al. 2015 [108] | Case report | 63-year-old female with Crohn’s disease | USA | 2011 | 1 | Not Applicable | Any adverse effects |  | 1 |
| Nishioka Sde A et al. 1998 [109] | Cross-sectional | The study included 39 women who attended a university hospital with spontaneous abortion (cases) and 74 pregnant women attending the antenatal clinic of that hospital (controls). | Brazil | 1993 | 111 | Yes | The crude odds ratio (relative risk estimate) of this association was 2.49, which dropped to 2.29 (95% CI 0.65–8.03) when adjusted for several confounders by multiple logistic regression. Dengue and exposure to organophosphate insecticide fogging during pregnancy were not associated with spontaneous abortion. |  | 2 |
| Nordin JD et al. 2013 [110] | Cross-sectional | General population | USA | VSD (1991-2006); DoD (1999-2007) | 1,171,889 | Yes | allergic or local reactions; rare visceral events; only six inpatient neurologic events |  | 1–2 |
| Nortey E et al. 2025 [111] | Case report | 48-year-old male | Ghana | Not reported | 1 | Not Applicable | Atraumatic Splenic Rupture |  | 4 |
| Nzolo D et al. 2018 [112] | Cohort study | entire population subjected to mass vaccination campaign | Democratic Republic of the Congo | 2016 | 4020 | Yes | The most frequently reported systemic AEFIs were fever, headache and diarrhea whilst the most frequently reported local AEFIs were injection site pain and injection site reaction, mainly injection site swelling | The primary objective of this study was to describe the safety profile of YFV, as captured from community-based Pharmacovigilance. | 1–2 |
| Oliveira et al. 2020 [113] | Case report | 9 month-old infant | Brazil | Not Reported | 1 | Not Applicable | fever (38°C), chill, nausea, and vomiting, strabismus, lack of control of cervical musculature and reduction of muscle strengths in lower limbs | Recovered | 2 |
| Otshudiema et al. 2017 [114] | Cohort study | Persons aged ≥9 months | Democratic Republic of the Congo | 2016 | 2.800 | Not Reported | 13 nonserious AEFIs: Cutaneous allergic reaction, rash, itching, Gastrointestinal syndrome, vomiting, fever, Injection-site pain and erythema, tiredness, muscle pain, Undetermined hematuria and tiredness, Allergic reaction on lips, Eye allergic reaction, conjunctivitis. 2 Serious: - Gastrointestinal syndrome, muscle pain, injection-site pain and erythema (severe malaria and urinary tract infection), - Spontaneous abortion of an unrecognized early pregnancy (endometritis). | Overall, 15 AEFIs were identified by active surveillance among approximately 2,800 patient records reviewed at the two targeted referral hospitals, including eight AEFIs previously reported during the immunization campaign. Two AEFIs were classified as serious and 13 as nonserious | 1–4 |
| Pereima RR et al 2022 [115] | Case series | 2 adults and 2 children | Brazil | 2017-2019 | 4 | Not Applicable | Case 1 presented with typical findings of central serous chorioretinopathy which resolved spontaneously; case 2 was diagnosed with acute Vogt-Koyanagi-Harada disease; cases 3 and 4 had bilateral diffuse retinal vasculitis |  | 2–3 |
| Perumalswami P et al. 2009 [116] | Case report | Women 31 years | USA | Not reported | 1 | Not applicable | 31- years woman with severe autoimmune hepatitis apparently triggered by hepatitis A or yellow fever vaccination |  | 4 |
| Pires-Marczeski FC, et al. 2011 [117] | Case series | Adults | Argentina | 2008 | 2 | Not Applicable | headache, fever, and malaise, expression aphasia and mnestic disorders, paraparesia, urinary retention, constipation, symmetric proximal weakness of the lower extremities, diffuse periventricular hyperintensity (RM) | The results of this work would indicate blood–brain barrier damage only for case 1. For case 2, the blood–brain barrier was not disturbed, indicating that the YFV-specific IgM detected in CSF was locally synthesized. | 3 |
| Pistone et al. 2010 [118] | Cross-sectional | HIV+ | France | 2002-2003 | 23 | Not Reported | Mild adverse events were reported in 4 patients. The adverse events were asthenia (n = 2), headache (n = 1), febricula (n = 1). No seriouas dverse events or cases of YF were recorded. |  | 1 |
| Pulendran B et al. 2008 [119] | Case report | Adults | USA | 2004 | 1 | Not Applicable | 5-day history of fever, chills, urinary frequency, and 1 day of nausea, vomiting, and diarrhea. a mild erythematous rash on his legs and trunk and a petechial rash on his face. AST, ALT, and direct bilirubin) peaked (at 318 IU/L, 82 IU/L, and 3.3 mg/dL, respectively), and the platelet count reached its lowest value, 30,000 cells/mm3. | These data suggest that prolonged viral persistence is highly unusual and possibly associated with the adverse events observed in this patient. | 2 |
| Rabello A et al. 2002 [120] | Cohort study | Adults from the staff of a private hospital | Brazil | 1999 | 14 | Not applicable | fever, headaches, body pains and muscular weakness and local reactions on the site of injection; rash, urticaria and asthma, encephalitis, multiple organ system failure and hepatitis |  | 2–5 |
| Raison-Peyron N et al. 2016 [121] | Case report | Man 29 years | Senegal | Not reported | 1 | Not Applicable | 29 years man with urticaria localized on cold exposed areas after vaccination for hepatitis A and yellow fever |  | 2 |
| Ramírez-Giraldo RH et al. 2024 [122] | Cross-sectional | Persons with history of egg allergy | Colombia | 2014-2023 | 171 | Yes | All patients tolerated the application of YFV without developing hypersensitivity reactions | Specific study on anaphylactic shock in subjects with a history of egg allergy | 1 |
| Ribeiro AF et al. 2021 [123] | Cross-sectional | Patients with YF vaccine–associated neurologic disease | Brazil | 2017-2018 | 50 | Not Applicable | 32 had meningoencephalitis (14 with reactive YF IgM in cerebrospinal fl uid), 2 died, and 1 may have transmitted infection to an infant through breast milk. Of 7 cases of autoimmune neurologic disease after YF vaccination, 2 were acute disseminated encephalomyelitis, 2 myelitis, and 3 Guillain-Barré syndrome |  | 3–5 |
| Rolfes et al. 2019 [124] | Case report | Adults | Germany | 2018 | 1 | Not Applicable | etraparesis, conjugated gaze palsy, aphasia, and dysphagia in patient with relapsing-remitting multiple sclerosis that assumes Fingolimod |  | 3 |
| Rowland M et al. 2012 [125] | Case report | 21-year-old healthy male | USA | 2012 | 1 | Not Applicable | right lower quadrant abdominal pain, subjective fevers, nausea, myalgia, anorexia |  | 2 |
| Rutkowski K et al. 2013 [126] | Case series | Adults and Children (2-8 years) | UK | 2006-2012 | 6 | Not Applicable | local erythema/ angioedema at the injection site, fleeting generalized urticaria, | Egg allergy had been diagnosed in early childhood in all subjects | 1–2 |
| Sako EY et al. 2014 [127] | Case report | Woman 56 years | Not reported | Not reported | 1 | Not Applicable | Injection site reaction followed by a localized bullous fixed drug eruption after the yellow fever vaccine |  | 2 |
| Schöberl F et al. 2017 [128] | Case report | 23-year-old male | Germany | Not Reported | 1 | Not Applicable | Manifestation of Neuromyelitis Optica Spectrum Disorder (NMOSD) with a typical postrema-area syndrome after yellow fever vaccination; and (B) unusual painful segmental erythema due to a dorsolateral spinal cord lesion at C6/7 level. |  | 3 |
| Scott RM et al. 1983 [129] | Cohort study | Volunteers >18 and <56 years | USA | Not reported | 38 | Yes | leukopenia (55%), macular rash (15%), fever (10%). |  | 1–2 |
| Sidibe et al. 2012 [130] | Cohort study | HIV+ | Mali | 2008 | 115 | Yes | Only non-serious AEFIs were reported at least once by 27 patients (24%). Fever was the most common minor adverse event, reported by 20 patients (17%), followed by local injection site reactions (5 patients), headache (2 patients), malaise (2 patients), flu-like symptoms (1 patient), urticaria (2 patients), herpes zoster (1 patient) and other, non-specific symptoms (2 patients). |  | 1 |
| Silva ML et al. 2010 [131] | Case report | Adults | USA | 2006 | 1 | Not Applicable | prominent encephalitis and a residual tongue tremor as- sociated with an important case of pancreatitis and rhabdomy- olysis. |  | 3 |
| Stangos A et al. 2006 [132] | Case report | 50-year-old woman | Switzerland | 2006 | 1 | Not Applicable | left-eye visual loss, associated with photopsias and a para-central scotoma |  | 3 |
| Struchiner CJ et al. 2004 [133] | Case series | A 19-year-old woman and a 4-year-old boy | Brazil | 2001 | 2 | Not Applicable | YF-vaccine associated viscerotropic disease: haemorrhagic fever and sepsis and rostration, vomiting, anorexia and abdominal pain. |  | 5 |
| Tanizaki et al. 2016 [134] | Cohort study | Healthy adults | Japan | 2011-2012 | 666 | Yes | Total AEs 55.6%, Systemic AEs 38.5%, Local AEs 34.5%, Both Systemic and Local AEs 17.7%. | Comparison between travellers of <60 years and ≥ 60 years | 1–2 |
| Tanos Lopes FT et al. 2023 [135] | Cross-sectional | Children with suspected egg allergy | Brazil | 2018-2020 | 435 | Yes | A total of 414 (95.2%) children had no vaccine reactions. Of the 21 (4.8%) children who had some reaction, 10 experienced a local reaction, 9 a mild skin reaction distant from the vaccine site, 1 presented local cutaneous reaction distant to the vaccination site, and 1 patient developed possible anaphylaxis |  | 1–2 |
| Tiwari P et al. 2016 [136] | Cohort study | Travellers | India | 2012-2013 | 849 | Yes | Fever, headache, malaise, local pain |  | 1 |
| Troillet N et al. 2001 [137] | Case report | Man 50 years | Switzerland | Not reported | 1 | Not Applicable | 50 years man develope symptoms after yellow fever vaccination |  | 2 |
| Valim V et al. 2020 [138] | Cohort study | Individuals of both sexes, aged from 18 to 88 years with autoimmune diseases (AID) and healthy controls (HC) | Brazil | 2017 | 278 | Yes | The analysis of local and systemic AE did not reveal significant differences in AID patients relative to HC (8 vs. 10% and 21 vs. 32%; p = 1.00 and 0.18, respectively). | A total of 249 clinical records, including 211 from patients with AID and 38 from HC, were obtained by interview and patient diary reports. Local AE included: pain, pruritus, hyperemia, edema, or node at the application site; Systemic AE included: fever, headache, myalgia, arthralgia, weakness, tremor, urticaria, angioedema, anaphylactic reaction, jaundice, peripheral edema. | 1 |
| van de Pol EM et al. 2014 [139] | Case report | 38-year-old man | Netherlands | 2013 | 1 | Not Applicable | Malaise, headache, arthromyalgia, nausea, and abdominal pain, jaundice, and abnormal laboratory findings |  | 3 |
| Vasconcelos PF et al. 2001 [140] | Case series | 5-year-old white girl and 22-year-old black woman | Brazil | Not Reported | 2 | Not Applicable | fatal cases of haemorrhagic fever associated with yellow fever | These serious and hitherto unknown complications of yellow fever vaccination are extremely rare, but the safety of yellow fever 17DD vaccine needs to be reviewed | 5 |
| Veit et al. 2009 [141] | Cohort study | HIV+ | Switzerland | 1996-2005 | 102 | Yes | No serious AEs was found (0%) | This article only analyzes severe cases. | 1 |
| Vellozzi et al. 2006 [142] | Cross-sectional | Adults | USA | 1996-2004 | 11 | Not Applicable | 11 Yellow Fever Vaccine-Associated Viscerotropic Disease |  | 5 |
| Vignapiano R et al. 2022 [143] | Case report | 48-year-old man | Italy | 2020 | 1 | Not Applicable | Corneal graft rejection in the left eye 3 weeks after a yellow fever vaccination |  | 2 |
| Volkov et al. 2020 [144] | Case report | Adults | France | 2018 | 1 | Not Applicable | Viscerotropic disease and acute uveitis | The case of a 37 years old man, previously healthy, with no medical history, no treatment, and no travels abroad. | 5 |
| Wauters RH et al. 2017 [145] | Case report | 31 year old female | USA | Not reported | 1 | Not Applicable | Serosanguinous vesicles ,bullous lesions and circumferential hyperpigmentation |  | 2 |
| Werfel U et al. 2001 [146] | Case report | Man 71 years | Not reported | Not reported | 1 | Not Applicable | Sistemic inflammatory reaction that began 5 days after yellow fever vaccination |  | 2 |
| Whittembury A et al. 2009 [147] | Case series | Adults | Perù | 2007 | 5 | Not Applicable | Case 1 (Fever, headache, arthralgia, myalgia, malaise, nausea, vomiting, diarrhea 8 days after vaccination developed shock, ARDS, acidosis, encephalopathy, multi-organ failure); Case 2 (Fever, headache, malaise, myalgia, nausea, vomiting,diarrhea 11 days after vaccination shock, encephalopathy, acidosis, GI bleeding, jaundice, ARDS, multi-organ failure); CASE 3 (Fever, malaise, dyspnea, abdominal pain, vomiting, diarrhea 9 days after vaccination, progressive shock, ARDS, acidosis, renal failure); Case 4 (Headache, malaise, arthralgia. 29 days after vaccination hospitalized with generalized edema, jaundice, altered mental status, then developed bleeding, acidosis, cardio-respiratory distress); Case 5 (Fever, headache, malaise, diarrhea. Admitted to ICU with dehydration, scleral icterus, tachypnea. Defervesced and was discharged 16 days after vaccination). | four of the five died of con- firmed YEL-AVD | 5 (4 deaths) |

CTCAE: Common Terminology Criteria for Adverse Events

AEFIs=Adverse Events Following Immunization

AEs=Adverse Events

SAEs=Serious Adverse Events

**Table S2**. Quality assessment of the included studies, using the Newcastle-Ottawa Scale (NOS), reported cohort studies and cross-sectional studies

| **Author year [Ref]** | **Selection** | | | | **Comparability** | | **Outcome** | |
| --- | --- | --- | --- | --- | --- | --- | --- | --- |
|  | **Item 1** | **Item 2** | **Item 3** | **Item 4** | **Item 5a** | **Item 5b** | **Item 6** | **Item 7** |
| Biscayart et al. 2014 [43] | * | * | * | * | * | * | * | * |
| Breugelmans et al. 2013 [44] | * | * | * | * | * | * | * | * |
| Bühler S et al. 2020 [47] | * | * |  | * |  | * | * | * |
| Cavalcanti DP et al. 2007 [49] | * | * | * | * | * | * | * | * |
| Cunha MP et al. 2013 [52] | * | * | * | * | * | * | * | * |
| Distenhreft JIQ et al. 2024 [56] | * |  | * | * | * |  |  | * |
| Durbin et al. 2009 [60] | * | * | * | * | * | * | * | * |
| Facincani et al. 2016 [63] | * | * | * | * |  | * | * | * |
| Fitzner J et al. 2004 [64] | * | * | * | * |  | * | * | * |
| García-Paba MB et al. 2023 [66] | * | * | * | * | * | * | * | * |
| Gerhardt CMB et al. 2020 [68] | * | * | * | * | * |  | * |  |
| Gnanadurai R et al. 2022 [69] | * | * | * | * | * | * | * | * |
| Hall Cet al. 2020 [71] | * | * | * | * | * |  | * | * |
| Huber et al. 2018 [72] | * | * |  | * | * | * | * | * |
| Ishaku SG et al. 2021 [73] | * | * | * | * | * | * | * | * |
| Kelso JM et al. 1999 [75] | * | * | * | * | * | * | * | * |
| Kernéis et al. 2013 [76] | * | * |  | * | * |  | * | * |
| Khromava et al. 2005 [77] | * |  | * | * |  | * | * | * |
| Kityo C et al. 2018 [78] | * | * | * | * | * | * | * | * |
| Kuntz et al. 2018 [80] | * | * | * | * | * | * | * | * |
| Lara AN et al. 2021 [82] | * | * | * | * | * |  | * | * |
| Lawrence et al. 2004 [83] | * |  | * | * | * | * | * | * |
| Le Hir A et al. 2024 [84] | * | * | * | * |  | * | * | * |
| Ledlie S et al. 2022 [87] | * | * | * | * | * | * | * | * |
| Lee JY et al. 2009 [88] | * | * | * | * | * | * | * | * |
| Liang G et al. 2025 [91] | * |  | * | * | * |  | * | * |
| Lindsey et al. 2008 [92] | * | * | * | * | * | * | * | * |
| Lindsey et al. 2016 [93] | * | * |  | * |  | * | * |  |
| Lopes et al. 2018 [95] | * | * | * | * | * | * | * | * |
| Martins Rde et al. 2014 [97] | * | * | * | * | * | * | * | * |
| Martins RDM. et al. 2010 [98] | * | * | * | * | * |  | * | * |
| McClenathan BM 2024 [99] | * | * | * | * | * | * | * | * |
| Miranda LJC et al. 2020 [102] | * | * | * | * |  |  | * |  |
| Miyaji et al. 2013 [104] | * | * | * | * | * | * | * | * |
| Miyazato Y et al. 2022 [105] | * | * | * | * | * |  | * | * |
| Monath et al. 2005 [106] | * | * |  | * | * | * | * | * |
| Nishioka Sde A et al. 1998 [109] | * | * | * | * | * | * | * | * |
| Nordin JD et al. 2013 [110] | * | * | * | * | * |  | * | * |
| Nzolo D et al. 2018 [112] |  | * | * | * | * | * | * | * |
| Otshudiema et al. 2017 [114] | * | * | * | * |  | * | * | * |
| Pistone et al. 2010 [118] | * | * | * | * | * |  | * | * |
| Rabello A et al. 2002 [120] | * |  | * | * |  | * | * | * |
| Ramírez-Giraldo RH et al. 2024 [122] | * | * | * | * | * | * | * | * |
| Ribeiro AF et al. 2021 [123] | * | * | * | * | * | * | * | * |
| Scott RM et al. 1983 [129] | * | * | * | * | * | * | * | * |
| Sidibe et al. 2012 [130] | * | * | * | * | * |  | * | * |
| Tanizaki et al. 2016 [134] | * | * | * | * | * | * | * |  |
| Tanos Lopes FT et al. 2023 [135] | * | * | * | * | * | * | * | * |
| Tiwari P et al. 2016 [136] | * | * | * |  | * | * | * | * |
| Valim V et al. 2020 [138] | * | * | * | * | * | * | * | * |
| Veit et al. 2009 [141] | * |  | * | * |  | * | * | * |
| Vellozzi et al. 2006 [142] | * | * | * | * | * |  | * | * |

* Each star represents a high-quality criterion accomplished by the study
